# Implant irritation and removal rates in operatively treated multiple rib fractures: a 49-month follow-up study

**DOI:** 10.1007/s00068-024-02681-x

**Published:** 2025-01-24

**Authors:** Felix Peuker, Roelien A. Haveman, Roderick M. Houwert, Thomas P. Bosch, Ruben J. Hoepelman, Fabrizio Minervini, Frank J. P. Beeres, Bryan J. M. van de Wall

**Affiliations:** 1 Department of Trauma Surgery, University Medical Center Utrecht, Utrecht, The Netherlands; 2 Department of Orthopedic and Trauma Surgery, Cantonal Hospital Lucerne, Lucerne, Switzerland; 3 Department of Health Sciences and Medicine, University of Lucerne, Lucerne, Switzerland; 4 Department of Trauma Surgery, Leiden University Medical Center, Leiden, The Netherlands; 5 Department of Thoracic Surgery, Cantonal Hospital Lucerne, Lucerne, Switzerland

**Keywords:** Multiple rib fractures, Implant irritation, Implant removal, Rib fixation

## Abstract

**Purpose:**

Little is known about the prevalence, impact and change of the symptoms after implant removal due to irritation in multiple rib fractures. This study aims to explore these aspects to improve treatment decision-making.

**Methods:**

Data was collected from two hospitals in the Netherlands and Switzerland. The study included only adults with operatively treated multiple rib fractures, regardless of whether the fractures were flail or non-flail. The primary outcome was the incidence of implant removal due to irritation. Secondary outcomes included implant irritation not leading to removal, other postoperative complications, and remission rates after implant removal. These outcomes were assessed during a follow-up phone call using a standardized questionnaire.

**Results:**

Hundred-twenty patients were identified, with 83 (69.2%) completing the final follow-up after a median of 49 months (IQR 40–59). Twenty-five (30.1%) patients experienced implant irritation, of whom four (4.8%) got their implant removed. Two (2.4%) reported significant improvement, one (1.2%) moderate, and one (1.2%) no improvement of symptoms.

**Conclusion:**

Implant irritation in patients with multiple rib fractures is a common problem, even years after surgery, without guaranteed symptom improvement post-removal. These results provide an additional argument to be more selective in offering rib fixation to patients with multiple rib fractures in the first place.

**Supplementary Information:**

The online version contains supplementary material available at 10.1007/s00068-024-02681-x.

## Introduction

In the past few decades, there has been growing interest in rib fixation for multiple rib fracture patients [1]. However, the additional value of this surgical procedure remains a topic of debate. The inconclusive outcomes of rib fixation for multiple rib fractures, the current strain on healthcare resources and the risk for various peri- and postoperative complications, seem to justify some scepticism about the trend to operate on all multiple rib fractures [2, 3, 4].

A common argument used against rib fixation is irritation caused by the implants. Although common, little is known about its prevalence. It often necessitates additional removal surgery in hopes of alleviating symptoms. However, studies on implant irritation in patients with multiple rib fractures are rare, with most literature focusing on isolated rib fractures or other (extremity) fractures [5, 6, 7]. Additionally, as implant irritation can cause problems years after the initial surgery, the commonly used 1-year follow-up period is too narrow.

The present study aims to provide long-term insights into the rates of implant irritation, subsequent removal, and remission in patients with multiple rib fractures using data from a large international prospective study (OPVENT study) [2, 8].

## Methods

### Study design

This was a prospective study of patients included in the OPVENT database who underwent rib fixation for three or more rib fractures [2]. The initial OPVENT study compared operative to conservative treatment in patients with multiple rib fractures between 2018 and 2021 in six level-1 trauma centers in Switzerland and the Netherlands. In the OPVENT study, operative intervention was administered to patients exhibiting clinical flail chest or thoracic deformity. Additionally, patients unable to attain a Numerical Rating Scale (NRS) score of less than 5 despite oral, intravenous, and/or epidural analgesic therapy were also candidates for surgery. (Table 1) For patients lacking clear operative indications, the decision between conservative and operative treatment was determined by the geographical location of the trauma and differences in preferred treatment methods among the admitting hospitals and treating surgeons.

The present study included only the adults aged 18 years or older with three or more rib fractures, as confirmed by CT scan, treated operatively in two of the six level-1 trauma centers. Only two trauma centers were selected for this study, as they were found to have included the majority of operatively treated patients.

The study population was subdivided into two groups: flail and non-flail rib fractured patients. This was done not with intent to compare the two patient populations but rather because they represent a distinct type of patient. The flail-chest group predominantly consists of more severely injured polytrauma cases.

This study complied to the Strengthening the Reporting of Observational Studies in Epidemiology (STROBE) guidelines [9].

**Table 1:** Indications for rib fixation

Fixation indications, *n* (%)	Total
Flail chest	28
Persistent pain	26
Thorax deformity	12
Persistent pneumothorax	6
Other	9

### Baseline characteristics

The following patient and trauma characteristics were collected from the OPVENT database: age, sex, American Society of Anaesthesiologists (ASA) score, and smoking status. Injury-related characteristics were Injury Severity Score (ISS), the incidence of polytrauma, the number of rib fractures and the incidence of concomitant injuries (i.e. pneumothorax, hemothorax) [10, 11, 12]. Polytrauma was defined as an ISS≥16 [13]. Also collected was the incidence of complications (i.e. pneumonia, empyema, infection osteosynthesis material (OSM), and superficial wound infection). Pneumonia was defined as a lower respiratory tract infection requiring antimicrobial treatment. It is identified by clinical signs such as high temperature (> 38.5 °C), suspected infiltrate upon auscultation, purulent sputum, leucocytosis, and elevated C-reactive protein [14]. From the operation reports the number of fixated ribs was collected. Intraoperative data on the diversity of surgical approaches of rib fixation and additional thoracic surgery (i.e. video-assisted thoracoscopic surgery (VATS), insertion of thorax drainage) was gathered as well.

### Outcomes

For the current study, a telephonic follow-up survey was conducted between February and July 2023 by two independent researchers, FP for the Dutch population and RAH for the Swiss population. Patients were asked a standardised set of questions with regard to implant irritation and removal as employed and described in a study performed on the same topic in patients with clavicle plates [9]. In summary, patients were asked: (1) whether patients had their implant(s) removed, (2) what the reason was for removal (i.e. implant-related irritation, implant failure, non-union), (3) to what extent implant removal improved any symptoms, (4) if there were other complications (i.e. deep or superficial infection), (5) whether patients are considering removal and why (not). The researchers also reviewed medical files to check and identify any complications. Patients who were unattainable after five attempts were labelled as ‘missing case’.

The primary objective of this study was to determine the incidence of implant removal due to irritation. Implant irritation was defined as pain or discomfort at the surgical/implant site without evidence for non-union, infection or implant failure/loosening.

Secondary outcomes included implant irritation not leading to removal (including reasons), reinterventions for other reasons than previously described and all complications including non-union and deep/superficial infections. The distinction between superficial and deep infection was based on the requirement for soft tissue debridement. Symptomatic non-union was defined as CT-confirmed non-healed ribs at least six months after trauma, with clinical symptoms.

### Surgical procedure

The initial rib fixation was performed as described by Beks et al. in the OPVENT study protocol with precontoured locking plates and screws of DePuy Synthes (MatrixRIBTM Fixation System) [2]. The removal of an implant due to irritation was only considered after one year following the initial fixation. Implant removal was conducted as follows: (1) Administering 2 g of intravenous Cefazolin; (2) Making an incision along the previous scar(s); (3) Retracting the screws and releasing the plate(4) Investigating for any specific alternative reasons for irritation/pain; (5) Closing the incision in multiple layers. Patients were released within two days if their wounds were dry and their pain was tolerable. Patients were allowed to weight bear and move the upper extremity freely as tolerated.

### Statistical analysis

SPSS version 26 was used for conducting statistical analyses (ⒸIBM). Categorical variables are reported as counts with percentages (%), while continuous variables are presented as means and standard deviations (SD) for parametric data. For nonparametrically distributed data, continuous variables are reported as medians with interquartile ranges (IQR). To assess the distribution of continuous variables, we employed the Shapiro-Wilk test and Q-Q plots. The Mann-Whitney U test was employed to compare nonparametrically distributed variables for ordinal/continuous variables, whereas the Pearson’s chi-square test was used for dichotomous variables. Significance was set at a p-value of less than 0.05.

## Results

**Fig. 1:**
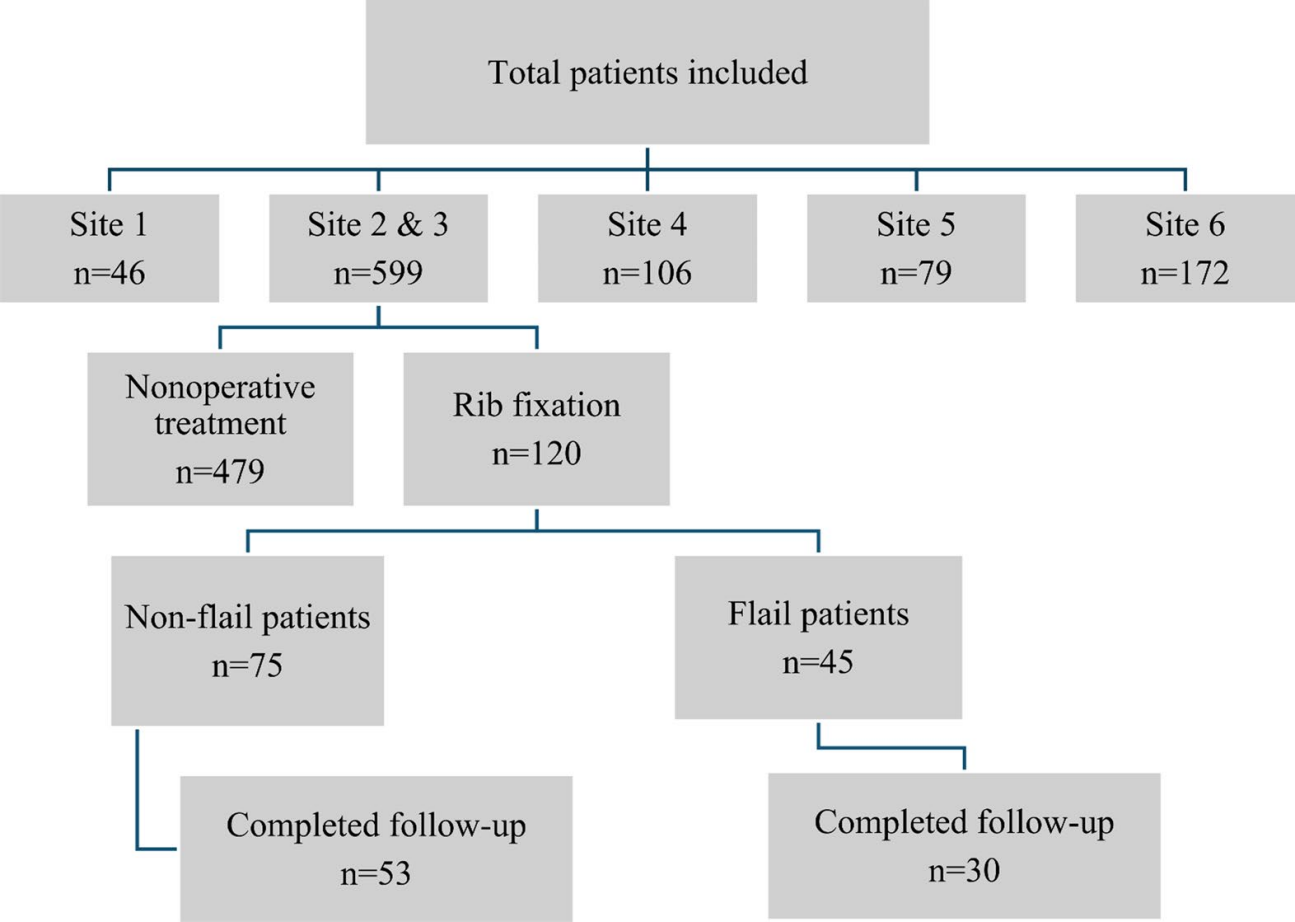
Flow chart of distribution

In total, 120 patients were identified from the OPVENT database with operatively treated multiple rib fractures between January 2018 and March 2021. Excluded from the study were 479 patients who received nonoperative treatment. Out of the 120 eligible patients, 75 had non-flail chest and 45 patients had flail chest injuries. 53 non-flail and 30 flail patients completed the final follow-up, consisting of a telephone survey (follow-up rate 69.2%; median follow-up period 49 months (IQR 40–59). Figure 1 shows a flowchart of the study population.

### Missing cases analysis

No differences in baseline characteristics were detected between included patients and patients that were lost to follow-up (Supplementary 1 & 2).

### Baseline characteristics

The baseline characteristics of all included patients are described in Tables 2 and 3.

### Primary outcome

Four patients (4.8%) underwent implant removal due to implant irritation. Implant removal for irritation was performed after a median interval of 29 months (range 28–47 months) after fixation (Table 4). Demographics of these four patients are described in Table 5.

### Secondary outcomes

At follow-up, 25 patients (30.1%) experienced implant irritation. Among them, seven patients (8.4%) considered implant removal, eight patients (9.6%) deemed the operation unnecessary, and six patients (7.2%) cited fear as the reason for not pursuing the procedure. Among the four cases (4.8%) that opted for removal, two (2.4%) reported significant improvement, one (1.2%) moderate, and one (1.2%) reported no improvement or deterioration of symptoms after removal).

Additionally, two more (2.4%) patients required implant removal within the first year due to implant failure. It is noteworthy that one (1.2%) patient claimed to have undergone implant removal at one of the admitting hospitals, however, no supporting documentation could be found, leading to the exclusion of this case from the analysis.

Throughout the entire follow-up period, no cases of fracture non-union were observed as a causative factor for pain in any patient. Additionally, one (1.2%) patient exhibited a superficial skin infection at the implant site at the final follow-up, while another patient (1.2%) experienced a deep infection subsequent to implant removal.

**Table 2:** Baseline patient and trauma characteristics^a^

Variables	Total	Non-flail chest patients	Flail chest patients
All, no. (%)	83	53	30
**Demographic data**			
Age at trauma, *mean* (SD)	62.0 (14.2)	62.3 (13.2)	61.4 (15.9)
Male sex, *n* (%)	65 (78.3)	42 (79.2)	23 (76.7)
ASA score, *n* (%)			
1–2	59 (71.1)	37 (69.8)	22 (73.3)
3–4	24 (28.9)	16 (30.2)	8 (26.7)
Smoker*, *n* (%)	16 (19.3)	9 (17.0)	7 (23.3)
**Injury-related characteristics**			
ISS, *median* (IQR)	19 (14–24)	17 (14–21)	25 (17–33)
Polytrauma (ISS≥16)*, *n* (%)	62 (74.7)	37 (69.8)	25 (83.3)
Number of rib fractures, *median* (IQR)	9 (7–11)	9 (7–11)	9 (8–14)
Bilateral rib fractures, *n* (%)	30 (36.1)	14 (26.4)	16 (53.3)
Isolated ventrolateral rib fractures, *n* (%)	13 (15.7%)	9 (17.0)	4 (13.3)
Dorsal rib fractures, *n* (%)	70 (84.3)	44 (83.0)	26 (86.7)
Concomitant injuries, *n* (%)			
Pneumothorax	70 (84.3)	41 (77.4)	29 (96.7)
Hemothorax	45 (54.2)	25 (47.2)	20 (66.7)
Sternum fracture	12 (14.5)	8 (15.1)	4 (13.3)
In-hospital complications, *n* (%)			
Pneumonia	19 (22.9)	13 (24.5)	6 (20.0)
Empyema	2 (2.4)	1 (1.9)	1 (3.3)
Infection OSM	0	0	0
Wound infection	1 (1.2)	0	1 (3.3)

^a^Abbreviations: ASA American Society of Anaesthesiologists, IQR Interquartile Range, ISS Injury Severity Score, OSM Osteosynthesis material, SD significant difference

**Table 3:** Baseline surgery characteristics^a^

Variables	Total	Non-flail chest patients	Flail chest patients
All, no. (%)	83	53	30
**Intraoperative data**			
Number of ribs fixated, *median (*IQR)	4 (3–5)	4 (3–4)	4 (3–5)
Surgical approaches, *n* (%)			
Anterolateral	4 (4.8)	3 (5.7)	1 (3.3)
Lateral	13 (15.7)	7 (13.2)	6 (20.0)
Posterolateral	28 (33.7)	22 (41.5)	6 (20.0)
Posterior	17 (20.5)	10 (18.9)	7 (23.3)
Combination	14 (16.9)	4 (7.5)	10 (33.3)
Additional thoracic surgery			
VATS	50 (60.2)	46 (86.8)	4 (13.3)
Thorax drainage	60 (72.3)	36 (67.9)	24 (80.0)

^a^Abbreviations: IQR Interquartile Range, VATS Video-assisted thoracoscopic surgery

**Table 4:** Incidence and indications for implant removal at follow-up (median follow-up 49 [40–59] months)

Variables,	Total	Non-flail chest patients	Flail chest patients
All, no. (%)	83	53	30
No implant irritation	58 (69.9)	36 (67.9)	22 (73.3)
Implant irritation	25 (30.1)	17 (32.1)	8 (26.9)
Removed	4 (15.4)	2 (11.1)	2 (25.0)
Not removed	21 (84.0)	15 (88.2)	6 (75.0)
No necessity despite irritation	8 (38.1)	6 (40.0)	2 (33.3)
No necessity due to fear	6 (27.3)	4 (25.0)	2 (33.3)
Removal in consideration	7 (31.8)	5 (31.3)	2 (33.3)

**Table 5:** Demographics and outcomes of the patients that underwent removal

Case number	Age	Sex	ASA	Number of rib fractures	Dislocated ribs	Dorsal fractures	Flail chest	ISS	Concomitant injuries	Number of ribs fixated	Time to removal	Improvement of symptoms
1	63	M	1	Right: ribs 3–7 Left: ribs 1–3	0	No	No	25	Hemopneumothorax, lung lacerations, lung contusion, scapula fracture	4	3 years	Considerable
2	55	M	2	Right: ribs 1–12 Left: ribs 1–9	6	No	Yes	33	Fracture parietal, temporal and zygomatic bone, hemopneumothorax both sides, long contusion, spleen laceration grade I/II	5	3 years	No
3	45	V	3	Right: ribs 1–9	1	No	No	19	C0/C1 vertebral fracture, Th1 proc. transversus fracture, long contusion	5	1 years	No
4	53	M	3	Right: ribs 1–11	3	Yes	Yes	21	Pneumothorax, scapula fracture both sides	2	1,5 years	Moderate

## Discussion

### Summary of results

Between January 2018 and March 2021 83 patients fulfilled final follow-up at a median of 49 months post-fixation. At that time point four patients (4.8%) eventually had their implants removed due to irritation. Additionally, twenty-one patients (25.3%) who still had the plate in place experienced implant irritation at final follow-up, of whom six (7.2%) did not proceed with implant removal out of fear and an additional seven (8.4%) considered implant removal but have not decided yet due to other reasons.

### Comparison to previous literature

To the best of our knowledge, only four studies have been conducted regarding patients with multiple rib fractures (including those with flail chest), and the occurrence of implant-related irritation and removal following rib fixation.

Peek et al. conducted a retrospective cohort study in which 74 out of the 102 rib fractured patients completed the follow-up questionnaires after a median follow-up period of 26 months (IQR 15–37) [5]. The median age of the participants was 62 years (54–75) and median ISS was 24 (20–29). In this study, the overall implant-related irritation exhibited a similar prevalence to our study, with 23 reported cases (31%). It, however, is worth noting that only two individuals from the entire cohort considered implant removal, but none of the 102 patients underwent implant removal. The ratio of intramedullary splints to osteosynthesis plates was not specified.

Furthermore, a study by Uchida et al. presented data and outcomes for a small group of 20 patients who were followed for an average of 47.5 months (IQR 22–58) [6]. Specifically, two patients (10%) experienced irritation symptoms related to the osteosynthesis plate. Notably, none of the patients chose to remove the implant during this extended follow-up period.

Moreover, Beks et al. conducted a study in which 103 out of 166 patients successfully completed the follow-up, over a median of 3.1 years (IQR 2.4–5.1) for flail chest patients and 4.4 years (IQR 3.4–5.9) for non-flail chest patients [7]. In this study, 83% of the patients underwent plate osteosynthesis. Notably, forty-nine (47.6%) patients reported experiencing implant-related irritation, and nine (8.7%) of these patients underwent implant removal due to irritation.

Hoepelman et al., using the same patient cohort from the OPVENT database, reported an implant irritation rate of 27% (*n* = 19) and a removal rate of 2.2% (*n* = 1.6) at the one-year follow-up. Our findings, with an irritation rate of 30.1% and a removal rate of 4.8%, suggest these rates remain stable even after a median follow-up of 49 months [2].

The studies mentioned above present varying rates of irritation and implant removal, which can be attributed to differences in follow-up duration, types of implants used, the definition of ‘implant-related irritation,’ and the specific survey questions employed. Also, cultural differences in the perception and coping of pain by patients as well as by physicians can play a considerable role [16].

Research on implant irritation and removal rates in cases of multiple rib fractures is limited. Our study is the only prospective study on this topic, also examining remission rates post-implant removal. Alongside the study by Peek et al., it stands as one of the few multicentre studies, enhancing its representativeness for the general population. Furthermore, our research includes the second largest known cohort of patients with multiple rib fractures (*n* = 83) and features one of the longest follow-up periods (49 months, IQR 40–59).

### Interpretation of results and clinical implications

The results of this study provide an additional reason to refrain or be more selective when deciding on initial rib fixation in patients with multiple rib fractures. Implant irritation is a considerable problem after rib fixation. Implants can cause irritation or pain in multiple ways, including chest tightness due to reduced flexibility, a foreign-body sensation from anterior plating under the skin and a thin layer of soft tissue, which can cause i.e. cold intolerance. Irritation or pain may also stem from intercostal nerve injury, which can lead to numbness or neuropathic pain [17, 18, 19]. Additionally, inflexible scar tissue can contribute to irritation.

In the present study 30.1% (*n* = 25) of patients experienced implant irritation to a varying degree leading to removal in 4.8% (*n* = 4) and in cases where removal was not performed, fear for reintervention (27.3%) or the choice for removal being under consideration (31.8%) was the leading argument.

Moreover, the removal of hardware material in general is considered unpredictable and can result in challenging surgeries due to bone overgrowth, screw head stripping and challenges in locating the material. This can potentially lead to complications like wound infection, nerve injury, increased blood loss, and pneumothorax [7, 18]. In our study, we observed only one patient (1.2%) with a deep infection after three years. However, considering that the need for rib fixation is potentially unnecessary, all complications caused by initial fixation and removal, are avoidable.

Not only the risk of complications, but also the outcomes on remission of complaints are unpredictable and not always as expected. Among the four cases that underwent removal, two (2.4%) reported significant improvement, one (1.2%) moderate, and one (1.2%) reported no improvement or even worsening of symptoms after removal. While the number of patients is too small to draw definitive conclusions, a wide range of outcomes is observed in this relatively small group. Unfortunately, there is limited literature available regarding remission rates after implant removal in (multiple) rib fractures [18].

An interesting finding is the fact that flail chest patients seemed to experience less implant irritation (26.9% vs. 32.1%) compared to non-flail patients. Logically, this is likely due to a higher rate of polytrauma and other injuries among flail chest patients that potentially overshadow implant irritation. However, it does emphasize that additional care should be taken when considering rib fixation in the non-flail group as the benefit of rib fixation for these particular patients is doubtful. For patients that do undergo rib fixation, multiple types of implants exist that theoretically should reduce the rate of irritation. These implants include intramedullary nails (IMN), intra-thoracic plates, titanium alloy plates and biodegradable plates. These implants should reduce irritation by either a reduced prominence or improvement in biocompatibility thus reducing scar tissue formation. Although some data exists proving their healing potential, no studies have yet been published comparing their ability to reduce irritation to that of traditional plates [15, 20, 21, 22, 23, 24].

Lastly, it is important to emphasize that the aetiology of implant-related complaints remains uncertain. Patients with conservatively treated rib fractures also seem to experience levels of chronic pain [25, 26]. Following a rib fracture, a localized inflammatory response is initiated, which can become chronic over time, also resulting in intercostal nerve damage and restrictive scar tissue. Baumann et al. previously explored long-term outcomes following rib fractures by comparing nonoperative and operative groups [26]. Their study found that 27.6% of nonoperatively treated patients reported some degree of pain after 17–20 months, compared to 28.2% in the operative group. A key question remains: is the long-term pain in operated patients primarily caused by the implant, or by the rib fracture itself?

### Limitations

There are several limitations that need to be considered. Firstly, we did not report Visual Analog Scale (VAS) scores for pain among patients to express the level of irritation/pain at follow-up. This decision was made because not all patients reported pain, but rather irritation, which in many cases was not the same. Additionally, a comprehensive measurement tool or gradation tool was lacking. Retrospectively determining pre- and post-operative VAS scores in the case of implant removal was also not feasible to the risk of recall bias. Instead, we utilized a Likert scale to objectify the change in symptom severity at follow-up.

Secondly, the study imposed a loss to follow-up of 30.8% after 49 months (IQR 40–59). Although this is a rather high percentage, the missing case analysis did not show any significant differences in baseline characteristics between the flail and non-flail group (Supplementary 1).

Thirdly, telephone follow-up was conducted by both a Dutch and Swiss investigator to facilitate effective communication with both Swiss and Dutch patients. Despite establishing a protocol with standardized closed questions beforehand to attempt to correct for potential inter-rater variability, complete avoidance of this variability was not possible.

Fourthly, the primary outcome measure—implant removal—has a low incidence (*n* = 4). As a result, the described percentage has a measure of uncertainty. Also, it made it impossible to relate any prognostic factors to the outcome of interest.

## Conclusions

A substantial number of patients with multiple rib fractures experiences implant irritation after fixation (30.1%) to a varying degree ultimately leading to removal in 4.8% (*n* = 4). After removal, improvement of these complaints is not guaranteed and involves surgical risks. These findings form an additional argument to be more selective in offering initial rib fixation in patients with multiple rib fractures.

## Electronic supplementary material

Below is the link to the electronic supplementary material.

Supplementary Material 1

Supplementary Material 2

## Data Availability

The data that support the findings of this study are not openly available due to reasons of privacy and sensitivity, but are available from the corresponding author upon reasonable request.
