# Supplementary figures and images for: Implant irritation and removal rates in operatively treated multiple rib fractures: a 49-month follow-up study
Source: Eur J Trauma Emerg Surg. 2025 Jan 24;51(1):76. doi: 10.1007/s00068-024-02681-x (PMC11761822; doi:10.1007/s00068-024-02681-x)

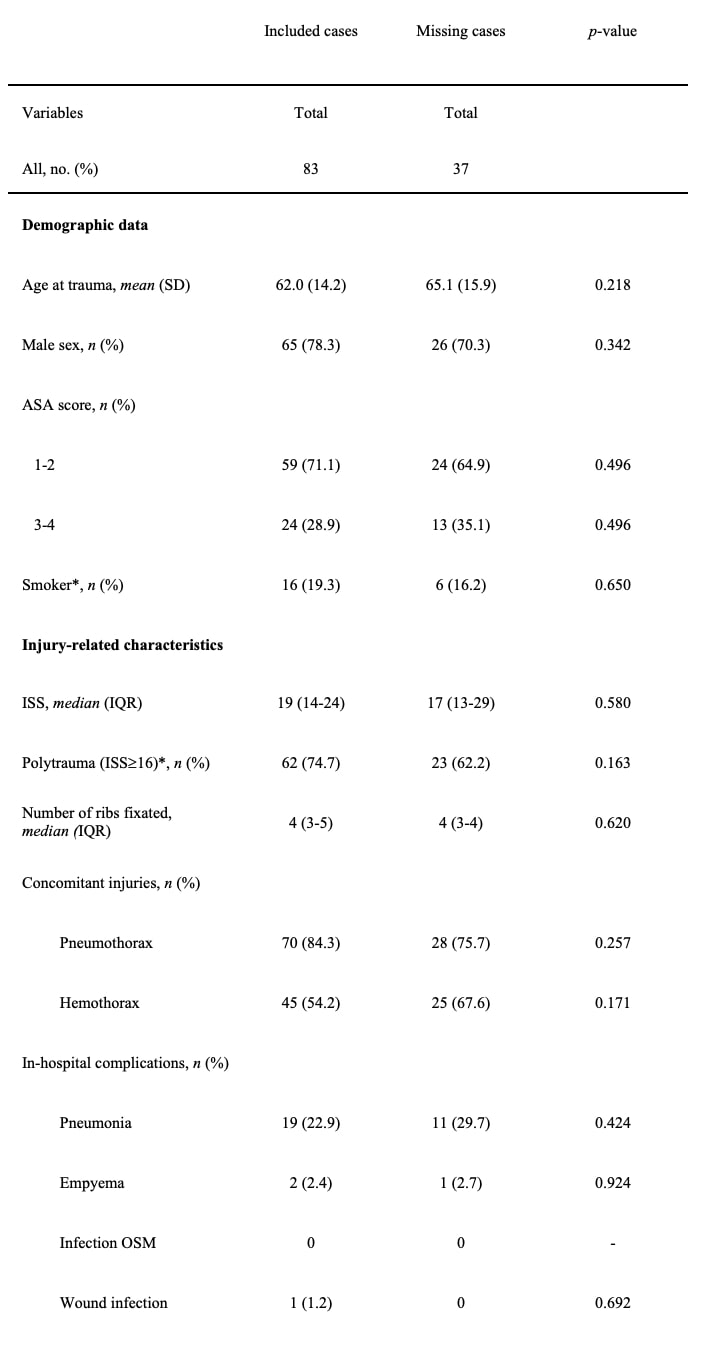

Supplement: Supplementary file 1 — Supplementary Material 1 [file 68_2024_2681_MOESM1_ESM.jpg]

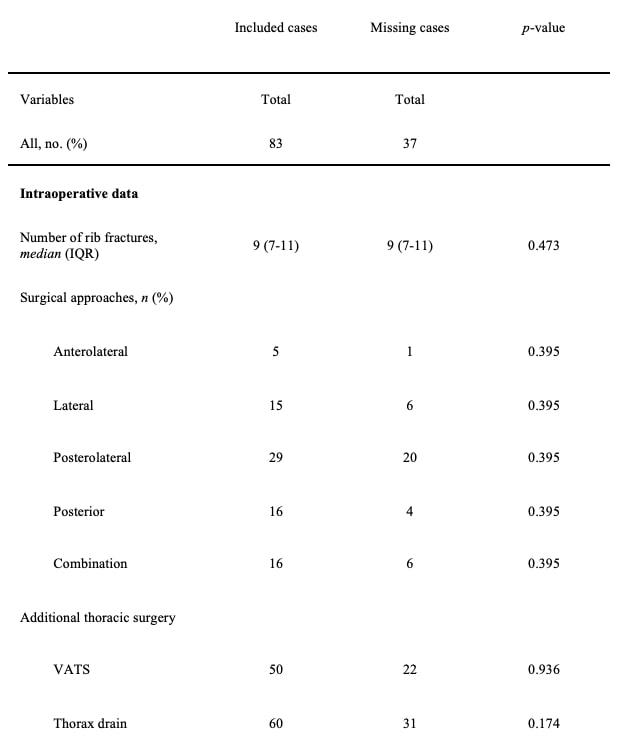

Supplement: Supplementary file 2 — Supplementary Material 2 [file 68_2024_2681_MOESM2_ESM.jpg]
